# Skin Ageing: Natural Weapons and Strategies

**DOI:** 10.1155/2013/827248

**Published:** 2013-01-29

**Authors:** Ivana Binic, Viktor Lazarevic, Milanka Ljubenovic, Jelena Mojsa, Dusan Sokolovic

**Affiliations:** ^1^Clinic of Dermatovenereology, Clinical Centre of Nis, Bovlevard Dr Zorana Djindjica 48, 18000 Nis, Serbia; ^2^Faculty of Medical Science, University of Kragujevac, Svetozara Markovica 69, 34000 Kragujevac, Serbia; ^3^Faculty of Medicine, University of Nis, Bovlevard Dr Zorana Djindjica 81, 18000 Nis, Serbia

## Abstract

The fact that the skin is the most visible organ makes us aware of the ageing process every minute. The use of plant extracts and herbs has its origins in ancient times. Chronological and photo-ageing can be easily distinguished clinically, but they share important molecular features. We tried to gather the most interesting evidence based on facts about plants and plant extracts used in antiaging products. Our main idea was to emphasize action mechanisms of these plant/herbal products, that is, their “strategies” in fighting skin ageing. Some of the plant extracts have the ability to scavenge free radicals, to protect the skin matrix through the inhibition of enzymatic degradation, or to promote collagen synthesis in the skin. There are some plants that can affect skin elasticity and tightness. Certainly, there is a place for herbal principles in antiaging cosmetics. On the other hand, there is a constant need for more evaluation and more clinical studies in vivo with emphasis on the ingredient concentration of the plant/herbal products, its formulation, safety, and duration of the antiaging effect.

## 1. Introduction 

The process of ageing begins at the moment we are born. The fact that the skin is the most visible organ makes us aware of the ageing process every minute. The eternal desire of people around the world is to live longer, to be young longer, or at least to look younger. We are socially, sexually, and physically active for an increasingly longer time [1]. Why should our wrinkles always remind us of the inexorable passing of time [1]? 

In the 21st century, the age of modern science and burst of technology, with plastic surgery and laser rejuvenation techniques, one question is imposing—is there a place for natural, herbal products? 

There is a growing tendency for physicians to use less invasive procedures that reduce the risks and complications. Patients not only wish to look younger but also want fewer scars [2]. 

Over the last decade, there has been an increase in scientific interest in reducing the appearance of ageing [3]. The use of plant extracts and herbs has its origin in ancient times, with the earliest records originating from ancient China and Egypt [4]. Plants produce a great variety of organic compounds and can be classified into three major groups: terpenoids, alkaloids, and phenolic compounds [5]. 

We tried to gather the most interesting evidence based on facts about plants and plant extracts used in antiaging products. Our main idea was to emphasize action mechanisms of these plant/herbal products, that is, their “strategies” in fighting skin ageing.

## 2. Chronological and Photo-Ageing 

There are two different types of changes that occur in the skin. Changes in the skin resulting from the passage of time alone are called chronological ageing. The term photoageing refers to the changes resulting from chronic sun exposure.

Clinical manifestation of chronologically aged skin includes xerosis, laxity, wrinkles, slackness, and the appearance of a variety of benign neoplasms such as seborrheic keratosis and cherry angioma [6]. Hair becomes depigmented, terminal hair converted to vellus hair, loss of hair is increased. There are changes in nail plate. There are fewer glands in aged skin [6]. 

The most evident and reproducible biological feature of ageing skin is the flattening of the dermal-epidermal junction [6, 7]. There is a general atrophy of the extracellular matrix [8], which is reflected by a decrease in the number of fibroblasts, reduced levels of collagen [8] and elastin, and their organization is impaired [7]. 

These changes are in part the result of cumulative endogenous damage from continuous formation of reactive oxygen species (ROS) generated during oxidative cell metabolism. Substantial evidence exists to support that ageing is associated with, though more likely, the consequence of free radical damage by various endogenous ROS [9]. There are in vivo evidence for a causal relationship between mitochondrial oxidative damage, cellular senescence, and ageing phenotypes in the skin [10]. 

Ageing is accelerated in areas exposed to sunlight (ultraviolet radiation), a process known as photoageing. It is called photoageing because of a combination of short wavelength (UVB) injury to the outer layers of the skin (epidermis) and long wavelength (UVA) injury to the middle layers (dermis) [11]. 

Clinical presentation of photoageing includes dryness of the skin, irregular pigmentation-freckles, lentigines, hyperpigmentation, wrinkling, and inelasticity [6]. Histologically there are an increased compaction of stratum corneum, increased thickness of granular cell layer, reduced epidermal thickness, elongation of epidermal rete ridges, and an increased number of hypertrophic dopa-positive melanocytes [6]. 

Ultraviolet radiation stimulates ROS synthesis, which has been implicated in mutagenesis and photoageing [9, 11]. 

Neutrophils are present in the sunburned skin. Moreover, they are potent producers of a wide array of proteolytic substances, including the pluripotent neutrophil elastase, Matrix metalloproteinase-8 (MMP-8), and MMP-9 [12]. 

Although the typical appearance of photoaged and chronologically aged human skin can be readily distinguished, recent evidence indicates that chronologically aged and UV-irradiated skin share important molecular features including altered signal transduction pathways that promote MMP expression [13], decreased procollagen synthesis, and connective tissue damage [14]. 

## 3. Free Radical Scavengers 

The free radicals are reactive chemical species that contain one or more unpaired electrons; they are products of oxidative cell metabolism [15]. The body and especially the skin are routinely exposed to stressful environmental factors such as pollutants and UV radiation, which produce a large number of aggressive oxidants that damage all the biological skin cell membranes [15]. 

A great number of plants and plant extracts are studied for their antioxidative action. Flavonoids like Rutin and phenolic compounds like Hesperidin derivates also have anti-tumor, antiviral and antibacterial activities, and antiradical and antioxidative activities [16]. The phenolic compounds are characterized by presenting in its chemical structure an aromatic ring linked to a hydroxyl group, which has a great ability to donate electron and hydrogen, this explains their exceptional antioxidant activities [15]. Among the characteristics of polyphenols of Green tea and Yerba Mate, the following deserves special attention: chemopreventive and therapeutic activities in cancer treatment; prevention of lipoperoxidation in mammals; prevention of adverse effects caused by UV radiation, with a reduction of oxidative damage and reduction in metalloproteinase production [15]. However, the topical use of tea compounds requires the solution of technical problems linked to the instability of catechins and their scarce penetration across the keratinized layer [17]. 

The extract of the fruits of the coffee plant (*Coffea arabica*) has shown to exhibit antioxidant activity mediated by potent antioxidant polyphenols, especially Chlorogenic acid, condensed proanthocyanidins, Quinic acid, and Ferulic acid [18, 19]. This extract showed improving fine lines, wrinkles, pigmentation, and overall appearance [18, 19]. Apigenin, a nontoxic botanical-derived fiavonoid occurring in numerous herbs, fruits, and vegetables, Curcumin obtained from the turmeric rhizome (*Curcuma longa*), Proanthocyanidins from the seeds of grapes (*V. vinifera*) [20], and Resveratrol, a polyphenol found in numerous plant species including grapes, peanuts, fruits, red wine, and mulberries, have also shown to possess the ability to protect the skin from harmful UV-induced effects by displaying antimutagen, antioxidant, free radical scavenging, anti-inflammatory, and anticarcinogenic properties [21]. 

Hormesis is a term used by toxicologists to refer to a biphasic dose response to an environmental agent characterized by low dose stimulation or beneficial effect, and a high dose inhibitory or toxic effect. In the fields of biology and medicine, hormesis is defined as an adaptive response of cells and organisms to a moderate (usually intermittent) stress. Examples include ischemic preconditioning, exercise, dietary energy restriction, and exposures to low doses of certain phytochemicals [22]. 

The hormetic induction of a stress response elicited by the hormetin Curcumin led to increased protection against a further oxidant challenge, supporting the view that mild stress-induced hormesis can be applied for the modulation of ageing and for improving the cellular functionality [23]. 

The ethanol extract of Licorice (*Glycyrrhiza glabra L*) showed powerful antioxidant activity by means of substantial ROS scavenging, hydrogen-donating, metal ion chelating, mitochondrial antilipid peroxidative and reducing abilities; the consequence was attributed to the high content of phenolic components [24]. 

Glycyrrhizin, a conjugate of one molecule of Glycyrrhetinic acid and two molecules of Glucuronic acid, is the main constituent of *G. glabra* [25]. It is considered to be the most common of the Asiatic folk medicines that acts as an anti-inflammatory agent on neutrophil functions including ROS generation [25]. Thus, Glycyrrhizin can be considered as a quenching agent of free radicals and a blocking agent of lipid peroxidation chain reactions. Tested on animal model, Glycyrrhizin showed an effective chemopreventive, antioxidant, and antiproliferative activity [25]. 

Extract of Mulberry (*Morus alba*) exhibited super oxide scavenging activity that is involved in the protection against autooxidation [26, 27]. 

The antioxidant activity of Basil, Oregano, and Thyme essential oils has been evaluated in a series of in vitro tests [28]. The antioxidant activity of Thymus species may be due to different mechanisms, such as prevention of chain initiation, decomposition of peroxides, prevention of continued hydrogen abstraction, free-radical scavenging [29], reducing capacity, and binding of transition metal ion catalysts [30]. Examples of new antioxidants within *Lamiaceae* species include phenolic diterpenes, phenolic carboxylic acids, biphenyls, and flavonoids extracted from Rosemary, Sage, Oregano, and Thyme [30]. 

There are data supporting potential beneficial effect of Poplar bud (*Populus nigra*) extract on skin ageing as it showed a strong modulation of transcription of genes involved in antioxidant defenses, inflammatory responses, and cell renewal [31]. 

*Illicium anisatum* essential oil has good antioxidant, anti-elastase, and anti-inflammatory effects and low cytotoxicity in human cell lines [32] (Table 1). 

## 4. Photoageing 

Photoageing and the development of skin cancer are of an increasing importance since changes in lifestyle have led to a significant increase in the individual commutative UV doses. In addition to the conventional organic-chemical and physical-mineral type sunscreens, other non-sunscreen protective strategies have been developed [33]. 

The extract of the tropical Cabbage palm fern (*Polypodium leucotomos*) is a plant-derived product that has been studied in vitro as well as in vivo. Its topical or oral administration is tolerated without toxicity. *P. leucotomos* demonstrates dual protective effects on the extracellular matrix via the inhibition of the proteolytic enzymes and the stimulation of TIMPs, structural collagens (types I, III, V) of extracellular matrix, and TGF-*β* in fibroblasts [34]. 

A review of numerous studies with Green tea (*Camellia sinensis*) has concluded that both oral consumption and topical application of green tea protects against inflammation and chemical- and UV-induced carcinogenesis. In addition, UV-induced immunosuppression is prevented by Green tea [35]. 

Silymarin, a fiavonoid complex isolated from the seeds of Milk Thistle (*Silybum marianum*), has been demonstrated to possess anti-inflammatory, antioxidative, and anticarcinogenic properties in vivo in animal models. Moreover, Silymarin may favorably supplement sunscreen protection and provide additional antiphotocarcinogenic protection [36]. 

It has also been shown that the Pomegranate (*Punica granatum*) extract protects human immortalized HaCaT keratinocytes against UVB-induced oxidative stress and markers of photoageing, and therefore, might be a useful supplement in skin care products [37]. The Catechin, an active component of *Punica granatum*, inhibited the UVB-induced skin photoageing [38]. 

Extract of *Ixora parviflora* attenuates UVB-induced photo damage and inflammation by modulating the expression of MMPs, Mitogen-activated protein kinases (MAPK) pathway, and Cyclooxygenase-2 (COX-2). This extract, therefore, appears to be a potent agent against the photoageing [39]. 

The Isoflavone extract from Soybean cake is a good candidate for an anti-photo-ageing agent in skin care. Furthermore, Isoflavone extract prevents skin cell apoptosis, erythema, and inflammation reactions [40]. 

It was recently shown that oral ingestion as well as topical application of rice wine suppresses epidermal barrier disruption caused by UV exposure [41]. Rice wine treatment decreased UV-induced epidermal thickening in mice. Based on these results, it is suggested that rice wine may actually be able to exert significant anti-ageing activities on skin. It has potential as an anti-ageing agent by stimulating pro-collagen synthesis, decreasing MMP-1 and tumor necrosis factor *α* (TNF-*α*) expression, and promoting laminin-5 production in skin cells as well as by reducing transepidermal water loss, skin wrinkling, and epidermal thickening in animal skin [42]. 

*Labisia pumila* extract clearly showed the photo protective potential [43] and could be used as an agent against extrinsic ageing. Apart from that, *Labisia pumila* could also upregulate the synthesis of collagen in human dermal fibroblast cells. The herbal extract also has the ability to protect the human skin from the ROS attacks generated by critical UVB exposure. This is mainly due to the presence of bioflavonoids and phenolic acids in the plant extract [44]. 

*Coffea arabica* extract, diminished UVB irritation induced photo-ageing by inhibiting MMPs and elevating type I pro-collagen production through ROS scavenging and downregulation of MAPK pathway [45]. 

Extract of *Emblica officinalis* fruit has an antioxidant activity related to UV protection (against photo-ageing) [46] (Table 2). 

## 5. Protection of the Skin Matrix 

A few years ago, an intriguing microinflammatory model of skin ageing was postulated, and offers an interesting approach to account for the loss of dermal elasticity and resilience as well as for wrinkle appearance [47]. Random tissue injury, for instance, as a result of ultraviolet exposure or the formation of reactive oxygen species, results in the development of a chronic vicious circle that in the course of time leads to increasing matrix damage. Both low-dose and high-dose ultraviolet radiations induce several cytokines, among them very prominently TNF-*α*, through a post-translational mechanism. 

Interleukin-6 (IL-6) mediates collagenolytic effects by modulating the ultraviolet- and infrared radiation-induced stimulation of MMP-1. On the other hand, it has been widely accepted that TNF-*α* substantially impairs collagen synthesis in the human skin via TNF-R55 activation. 

Ultimately, the clinical result is a substantial deterioration of the connective tissue leading to the development of wrinkles and the loss of skin elasticity and firmness [48]. Subjects of this destabilization are collagen, hyaluronan, and glycosaminoglycans. 

There are two mechanisms of the action of *Arctium lappa*. Firstly, anti-inflammatory effects in terms of inhibition of IL-6 and TNF-*α* effectively and continually protect the extracellular matrix from subclinical, chronic tissue inflammation. Secondly, the profound stimulation of connective tissue metabolism (e.g., collagen and hyaluronan synthesis) regenerates the dermal structure [48]. 

Topical treatment with an *A. lappa* fruit extract offers an effective skin care regimen for mature skin. Matrix metabolism is significantly stimulated in vivo and wrinkles are visibly reduced. Arctiin counteracts the chronic inflammation in the ageing skin offering the first cosmetic treatment option that targets these subclinical processes in the ageing skin [48]. 

Immunohistochemical study of the effects of *Castanela asiatica* on wound healing showed that TGF-*β* and Induced nitric oxide synthase (iNOS) immune-reactivity were weaker and laminin and fibronectin immune reactivity were stronger in the collagenase ointment group than the *C. asiatica*. Still, this study does not negate matrix protective effects or promotion of collagen synthesis of *C. asiatica* [49].

Phenolic substance purified from *Areca catechu* has an anti-ageing effect by protecting connective tissue proteins. The CC-517 was identified as a phenolic substance by using various specific methods. A remarkable inhibition of elastase by CC-517 may protect the major proteins of the extracellular matrix, activate its reconstruction, and indirectly improve the tone of the capillary walls [50]. 

Xanthorrhizol in *Curcuma xanthorrhiza* suppressed UVB-induced MMP-1 expression and increased type I pro-collagen expression [51]. *Polypodium leucotomos* extract has been shown to inhibit the activities of MMP-1, and MMP-2 [34], erythrodiol-3-acetate isolated from *Styrax japonica* inhibited UV-induced MMP-1 expression [52]. 

The Soybean Bowman-Birk inhibitor (BBI) is a water-soluble protein, a metalloprotein and the removal of metal bound to BBI enhances BBI inhibitory activity against MMP-1 [53]. 

The anti-inflammatory properties of the plant Wild Yam (*Dioscorea villosa*) make it suitable for dermatologic products used in the treatment of irritated or aged skins [54]. The extract also shows anticollagenase activity, suggesting a possible use in anti-ageing products and, in general, to fight skin degenerative syndromes [54]. Together with the advantageous effects of Diosgenin on ageing skin, it is suggested that Diosgenin may be a good and safe health food for the aged, especially to alleviate the effects of climacteric issues [55]. However, the effects of Diosgenin may differ dependently on the endogenous estrogen levels, tissue or cell types, the route of administration, the time, and level of exposure [55]. 

The root of Astragalus (*Radix astragali*) is one of the most popular Chinese herbs, which is used traditionally to strengthen the immune system, boost the energy, and promote skin health. Bacillus subtilis natto-fermented *Radix astragali* significantly stimulate hyaluronic acid production in cultured human epidermal keratinocytes and human dermal fibroblasts. The enhancement was not based on the growth stimulation of the skin cells, but corresponded well to the higher expression of hyaluronan synthetase transcripts [56]. 

*Camellia japonica* oil can induce the synthesis of type I collagen, has high moisturizing effect, and is safe to use. This suggests that Camellia japonica oil might be introduced as a possible antiwrinkle agent for the management of skin ageing [57]. 

It is demonstrated that *Panax ginseng* root extract can induce the synthesis of type I collagen, and the mechanisms underlying its action may be mediated via the Smad activation pathway. The Smads are a series of proteins that perform downstream functions from the serine/threonine kinase receptors of the TGF-*β* family [58]. 

The most interesting, according to our opinion, is the first demonstration of the cinnamon extract effect on human dermal fibroblasts. Cinnamon extract significantly promotes type I collagen biosynthesis within dermal fibroblasts. Cinnamaldehyde is the major active component in the Cinnamon extract that induces type I collagen biosynthesis. The underlying molecular mechanism is believed to trigger the activation of Insulin-like growth factor-I (IGF-I) signalling via the direct IGF-I receptor activating pathway. These findings could be helpful in improving the signs and symptoms of the ageing skin [59]. 

Amla extract (*Emblica officinalis*) elevates the mitochondrial activity of human skin fibroblasts and promotes production of procollagen. These results suggest that Amla extract has a number of potential mitigative, therapeutic, and cosmetic applications. [60] (Table 3). 

## 6. Depigmentation 

Many plant extracts are more potent inhibitors of melanin formation than Hydroquinone, Kojic acid or Arbutin, and are not associated with cytotoxicity or mutagenicity of melanocytes [61]. Whiter skin appears younger. 

The Licorice extract is the safest pigment-lightening agent with the fewest side effects. The main ingredient in the hydrophobic fraction of licorice extract is glabridin, which inhibits tyrosinase activity in cultured B16 murine melanoma cells without affecting DNA synthesis. Glabrene, Isoliquiritigenin licuraside, Isoliquiritin, and Licochalcone A are other active compounds within licorice extract that inhibit tyrosinase activity. Liquiritin is another main active ingredient of Licorice extract, and it appears to induce skin lightening by dispersing melanin [20]. 

Raspberry ketone from *Rheum officinale* inhibits melanogenesis through a posttranscriptional regulation of tyrosinase gene expression in cultured B16 melanoma cells. In addition, Raspberry ketone also inhibits melanogenesis of the skin in both zebra-fish and mice; raspberry ketone would appear to have high potential for use in the cosmetics industry [62]. Tiliroside, an organic compound from raspberry, could significantly inhibit intracellular tyrosinase activity and melanin production. This evidently supports the idea that Tiliroside could be a potential skin whitening agent in cosmetic or pharmaceutical industries [63]. 

Arbutin, a naturally occurring b-D-glucopyranoside derivative of Hydroquinone, exists in the dried leaves of certain plant species, such as Bearberry (*Arctostaphylos uva-ursi*) and *Oryganum majorana* [64]. 

Deoxyarbutin (4-[tetrahydrofuran-2-yl-oxy]-phenol) has also demonstrated effective inhibition of mushroom tyrosinase in vitro. In a human clinical trial, topical treatment with deoxyarbutin for 12 weeks resulted in a significant or a slight reduction in overall skin lightness and improvement of solar lentigines in a population of light-skinned or dark-skinned individuals, respectively [65]. 

Origanoside from *Origanum vulgare* has a depigmentation effects, a fact that may be exploited in future food additives and skin-whitening cosmetics. The mechanism by which origanoside inhibits melanin synthesis results in the decline in cellular Dihydroxyphenyl-alanine oxidase (DOPA oxidase) activity, rather than in direct inhibition of tyrosinase activity. This phenomenon is associated with the down regulation of the gene and protein expressions of Microftalmia-asociated transcription factor (MITF), tyrosinase, and tyrosinase-related proteins 2 (TRP-2) of Origanoside in vitro and in vivo, which may result in a prominent antimelanogenic effect [61]. 

Aloesin, a compound isolated from the *Aloe vera,* has been proven to competitively inhibit tyrosinase from human, mushroom, and marine sources [66]. 

Mulberroside F (moracin M-6, 30-di-O-beta-D-glucopyranoside), the active component of Mulbery (*Morus alba*), shows inhibitory effects on tyrosinase activity and on melanin formation in melan-*α* cells, suggesting a role for *Morus alba* as a component of lightening cosmetics [66]. Mulberroside A was isolated from the ethanol extract of Morus alba roots. Mulberroside A, Oxyresveratrol, and Oxyresveratrol-3-O-glucoside showed depigmenting effects in brown guinea pig skin stimulated by UVB irradiation [67]. 

Treatment with *Radix ginseng* in the presence of various concentrations of *Radix trichosanthis* suppressed tyrosinase activity and melanin content but increased cell proliferation slightly in B16 melanoma cells, raising the possibility that this combination may be effective as a skin-lightening agent [68]. 

Diosgenin from Wild Yam (*Dioscorea villosa*) extract has a depigmenting effect and can therefore be used in melasma, melanodermatitis, and sun lentigo [69]. A study carried out on melanoma cells has shown that the depigmenting effect is linked to the activation of the cellular Phosphatidylinositol-3-kinase (PI3K) pathway, suggesting that Diosgenin may be an effective inhibitor of hyperpigmentation [69]. 

Methanol extract of *Eupatorium triplinerve Vahl* demonstrated inhibitory activities on melanin formation in B16 melanoma cells and tyrosinase enzyme activity [70]. 

There are eighteen known phenolic compounds and two sulfur containing compounds isolated from pineapple fruit *Ananas comosus*. These compounds may contribute to the well-known antibrowning effect of pineapple juice and may be potential skin whitening agents in cosmetic applications [71]. 

Saponified Evening Primrose oil (*Oenothera biennis*) exerts a pigment-whitening effect by inhibiting the expression of tyrosinase and related enzymes; therefore, this effect may be related to the high proportions of Linoleic acid released by saponification from Evening Primrose oil [72]. 

Persimmon (*Diospyros kaki*) leaf extract demonstrated anti-wrinkle and skin-lightening effect comparable to that of hydroquinone effect, without any side effects [73]. 

Proanthocyanidins contributed greatly to the melanogenesis-inhibiting effect of Rose hips extract (*Rosa canina*) in B16 mouse melanoma cells. Moreover, Rose hips extract inhibited pigmentation together with tyrosinase activity in guinea pig skin. These data suggest that Rose hips extract may be useful as a skin whitening agent when taken orally [74]. 

Gingerol, is an active component of Ginger (*Zingiber officinale*), shows antipyretic and anti-inflammatory activities [75]. It inhibits melanin synthesis in murine B16F10 melanoma cells, by reducing MITF and inhibiting the tyrosinase activity [75] (Table 4). 

## 7. Elasticity and Tightening 

Ethanol extracts of *Glycyrriza glabra, Curcuma longa* (roots], seeds of *Psorolea corylifolia*, *Cassia tora, Areca catechu, Punica granatum,* fruits of *Embelica officinale*, leaves of *Centella asiatica,* dried bark of *Cinnamon zeylanicum,* and fresh gel of *Aloe vera* in varied concentrations showed improvement of the viscoelastic and hydration properties of the skin. These beneficial effects might be due to the synergistic antioxidant, anti-inflammatory and UV protective properties of the herbal ingredients [76]. 

Hops (*Humulus lupulus L*) extracts are also useful in the prevention of skin ageing and in the treatment of loose skin, stretch marks, and sagging [77]. They are also used in breast tightening to promote the presence of the phytoestrogen 8-prenylnaringenin, even though clinical trials do not allow a reliable evaluation of this treatment and cannot exclude the insurgence of noxious effects [55]. 

In certain African populations, women grind the fruit of Sausage Tree (*Kigelia Africana*) to a poultice, which is then spread on the breast to improve its firmness [55]. The fruit active principles are known to induce a firming effect on the dermis and its musculature. Such an effect would be due to isoflavones and steroid saponosides present in the fruit. Isoflavones are phytoestrogens acting on tissues in a way similar to that of human estrogens. Saponosides induce skin drainage and exert restitution, thus restoring the elasticity and firmness of the dermis [55]. 

Wild Yam's (*Dioscorea villosa*) Diosgenin is also used for breast cosmetic lifting since it seems to induce an increase of adipocyte volume resulting in an increase of breast turgor [55]. 

After treatment with the Dill (*Peucedanum graveolens*) extract, skin elasticity was improved, the skin felt more elastic, wrinkles appeared smoothed, and face contours appeared remodelled [78]. 

*Zanthoxylum bungeanum* is a functional cosmetic ingredient for the temporary improvement of skin wrinkles [79] (Table 5). 

## 8. Conclusion 

Phenolic compounds, Flavonoids, and Proanthocyanidins from plants are responsible for antioxidative activities of herbal products. This is explained by their chemical structure and their ability to donate free electron and hydrogen [15, 20]. 

The extracts of the tropical Cabbage palm fern (*Polypodium leucotomos*) [34] and Green tea (*Camellia sinensis*) have strong photoprotective properties [35]. The great number of plant extracts can diminish UVB-induced photo damage by decreasing activity of enzymes involved in tissue degradation (i.e., *Ixora parviflora, Coffea arabica*) [39, 45], or by increasing of synthesis tissue constituents (i.e., *Labisia pumila*) [43].

Numerous plants and plants extracts can attenuate degradation of skin matrix.* A. lappa *[48], *A.catechu *[50], *D. villosa *[54], *C. xanthorrhiza *[51], and *S. japonica *[52] are examples of plants that can inhibit Hyalorunidase, Elastase, Colagenase, and MMP. Some plants have the ability to promote synthesis of collagen, that is, *E. oficinalis *[60], *C. asiatica *[49], *P. ginseng *[58], and *C. zeylanicum *[59]. 

Plants and plant extracts with depigmentation properties act through various mechanisms: inhibition of melanogenesis [62], dispersion of melanocytes [20], inhibition of Tyrosianase [62, 63], decline in activity of cellular DOPA oxidase [61], and downregulation of the gene and protein expression of the MITF [61].

Some plants can improve skin firmness and elasticity, mainly due to phytoestrogens and saponosides [55]. 

Plant extracts are often considered safe [33], because of the simple fact that they come from nature [80]. On the other hand, irritation, contact allergic dermatitis, and other adverse reactions to natural products have been documented [55, 81].

Over the past decade, a great number of plant extracts have been studied. In our opinion, there is a constant need for more evaluation and more clinical studies in vivo with the emphasis on the ingredient concentration of the herbal products, their formulation, safety, and the duration of the anti-ageing effect.

## Figures and Tables

**Table 1:** Plants with antioxidative activity.

Antioxidative activity (free radicals scavenging)	
Grape	*Vitis vinifera *
Green Tea	*Camellia sinensis *
Tumeric	*Curcuma longa *
Mulbery	*Morus alba *
Antioxidative activity	
Licorice	*Glycyrrhiza glabra *
Japanese star anise	*Illicium anisatum *
Yerba Mate	*Ilex paraguariensis *
Coffee	*Coffea arabica *
Poplar bud	*Populus nigra *
Thyme	*T. caucasicus, T. kotschyanus *and *T. vulgaris *
Rosemary	*Rosmarinus officinalis *
Oregano	*Origanum vulgare *
Basil	*Ocimum basilicum *

**Table 2:** Plants with anti photo-ageing action.

UV protection	
Green Tea	*Camellia sinensis *
Pomegranate	*Punica granatum *
Cabbage palm fern	*Polypodium leucotomos *
Coffee	*Coffea arabica *
Milk thistle	*Silybum marianum *
Kacip fatimah	*Labisia pumila *
Photo-ageing	
Soybean	*Glycine max *
Coffee	*Coffea arabica *
Ixora	*Ixora parviflora *
Amla	*Emblica oficinalis *

**Table 3:** Plants with matrix protective action.

Antihyalorunidase activity	
Burdock	*Arctium lappa *
Areca nut palm	*Areca catechu *
Antielastase activity	
Areca nut palm	*Areca catechu *
Anticolgeenase activity	
Wild Yam	*Dioscorea villosa *
MMP inhibition	
Temulawak	*Curcuma xanthorrhiza *
Styrax	*Styrax japonica *
Cabbage palm fern	*Polypodium leucotomos *
Soybean	*Glycine max *
Coffee	*Coffea Arabica *
Stimulation of hyaluronic acid production	
Astragalus root	*Radix astragali *
Promotion of collagen synthesis	
Amla	*Emblica oficinalis *
Gotu Kola	*Centella asiatica *
Burdock	*Arctium lappa *
Camellia	*Camellia japonica *
Ginseng	*Panax ginseng *
Cinnamon	*Cinnamomum zeylanicum *

**Table 4:** Plants with lightening properties.

Licorice	*Glycyrrhiza glabra *
Rhubarb	*Rheum officinale *
Bearberry	*Arctostaphylos uva-ursi *
Ginseng	*Panax Ginseng *
Aloe	*Aloe vera *
Wild Yam	*Dioscorea villosa *
Japana Roxa	*Eupatorium triplinerve Vahl *
Oregano	*Origanum vulgare, O. majorana *
Pineaple	*Ananas comosus *
Evening primrose	*Oenothera biennis *
Persimmon	*Diospyros kaki *
Rose hips	*Rosa canina *
Ginger	*Zingiber officinale *
Raspberry	*Rubus ideus *
Mulbery	*Morus alba *

**Table 5:** Plants with tightening and firming action on the skin.

Tightening and firming action on the skin	
Licorice	*Glycyrriza glabra *
Tumeric	*Curcuma longa *
Psorolea	*Psorolea corylifolia *
Cassia	*Cassia tora *
Areca palm	*Areca catechu *
Pomegranate	*Punica granatum, *
Amla	*Embelica officinale *
Gotu Kola	*Centella asiatica *
Cinnamon	*Cinnamon zeylanicum *
Aloe	*Aloe vera *
Breast tightening	
Hops	*Humulus lupulus *
Sausage Tree	*Kigelia Africana *
Wild Yam	*Dioscorea villosa *
Skin elasticity	
Dill	*Peucedanum graveolens *
Sichuan pepper	*Zanthoxylum bungeanum *
